# The Acid Test of Fluoride: How pH Modulates Toxicity

**DOI:** 10.1371/journal.pone.0010895

**Published:** 2010-05-28

**Authors:** Ramaswamy Sharma, Masahiro Tsuchiya, Ziedonis Skobe, Bakhos A. Tannous, John D. Bartlett

**Affiliations:** 1 Department of Cytokine Biology, Forsyth Institute, and Department of Developmental Biology, Harvard School of Dental Medicine, Boston, Massachusetts, United States of America; 2 Division of Aging and Geriatric Dentistry, Tohoku University, Sendai, Japan; 3 Departments of Neurology and Radiology, Massachusetts General Hospital, and Program in Neuroscience, Harvard Medical School, Boston, Massachusetts, United States of America; East Carolina University, United States of America

## Abstract

**Background:**

It is not known why the ameloblasts responsible for dental enamel formation are uniquely sensitive to fluoride (F^−^). Herein, we present a novel theory with supporting data to show that the low pH environment of maturating stage ameloblasts enhances their sensitivity to a given dose of F^−^. Enamel formation is initiated in a neutral pH environment (secretory stage); however, the pH can fall to below 6.0 as most of the mineral precipitates (maturation stage). Low pH can facilitate entry of F^−^ into cells. Here, we asked if F^−^ was more toxic at low pH, as measured by increased cell stress and decreased cell function.

**Methodology/Principal Findings:**

Treatment of ameloblast-derived LS8 cells with F^−^ at low pH reduced the threshold dose of F^−^ required to phosphorylate stress-related proteins, PERK, eIF2α, JNK and c-jun. To assess protein secretion, LS8 cells were stably transduced with a secreted reporter, *Gaussia luciferase*, and secretion was quantified as a function of F^−^ dose and pH. Luciferase secretion significantly decreased within 2 hr of F^−^ treatment at low pH versus neutral pH, indicating increased functional toxicity. Rats given 100 ppm F^−^ in their drinking water exhibited increased stress-mediated phosphorylation of eIF2α in maturation stage ameloblasts (pH<6.0) as compared to secretory stage ameloblasts (pH∼7.2). Intriguingly, F^−^-treated rats demonstrated a striking decrease in transcripts expressed during the maturation stage of enamel development (*Klk4* and *Amtn*). In contrast, the expression of secretory stage genes, *AmelX*, *Ambn*, *Enam* and *Mmp20*, was unaffected.

**Conclusions:**

The low pH environment of maturation stage ameloblasts facilitates the uptake of F^−^, causing increased cell stress that compromises ameloblast function, resulting in dental fluorosis.

## Introduction

Fluoride (F^−^) at concentrations of 0.7 to 1.2 ppm in drinking water is beneficial as an anti-cariogenic [1]. However, higher levels of F^−^ can occur naturally in groundwater or on land, as is found in several areas in the world [2]. Chronic exposure to high dose F^−^ can result in dental fluorosis [3], skeletal fluorosis [4] as well as renal and thyroid toxicity [5]. However, the initial and most apparent effect of excess F^−^ is in dental enamel. Approximately 32% of children in the United States suffer from mild to severe forms of dental fluorosis [6], manifested as white spots of hypomineralized enamel to darkly stained and porous enamel that chips easily [7]. It is not known why tooth enamel is uniquely sensitive to F^−^.

Enamel formation occurs in stages. During the secretory stage, the enamel forming epithelial cells (ameloblasts) secrete large quantities of protein, including amelogenin, ameloblastin, enamelin and matrix metalloproteinase-20 (MMP-20). Together, these proteins form an organic matrix within which thin enamel ribbons of hydroxyapatite crystallize. The pH during the secretory stage of enamel formation is approximately 7.23 [8]. Once the enamel ribbons attain their full length, ameloblasts transition to the maturation stage. During this stage, ameloblasts secrete kallikrein-4 (KLK4) to degrade the matrix proteins and facilitate their resorption [9]. This allows enamel ribbons to grow in width and thickness and interlock to form mature hardened enamel [10]. Massive precipitation of hydroxyapatite mineral occurs during the maturation stage. Depending on the phosphate precursor, the creation of one mole of apatite releases 8–14 moles of H^+^ ions [11], [12]. Therefore, during the maturation stage of enamel development, ameloblasts are exposed to an acid environment that can dip below pH 6.0 [8].

We hypothesize that the low extracellular pH surrounding the maturation stage ameloblasts promotes the conversion of F^−^ to HF. When the pK_a_ value of HF (3.45) is substituted in the Henderson-Hasselbalch equation *(pH = pK_a_+log [F^−^]/[HF])*, we observe that at pH 7.4, the *[F^−^] ∶ [HF]* ratio is 8913∶1. However, at pH 6.0, this ratio decreases to 355∶1. Therefore, approximately 25-fold more HF is formed at pH 6.0 as compared to pH 7.4. While the exact concentrations of HF in the extracellular milieu may vary according to the level of water content and the presence of ions that could interfere with HF dissociation, the Henderson-Hasselbalch equation indicates that the concentration of HF increases as the pH falls. Unlike F^−^, HF can diffuse easily into the cell cytosol. Because the cytosol has a neutral pH, virtually all HF reverts to F^−^ and F^−^ cannot easily diffuse out of the cell. Therefore, if the pH of the extracellular matrix is lower than that of the cell cytoplasm, an intracellular-extracellular pH gradient is maintained that continuously drives HF into the cell. Over the course of months to years, the F^−^ concentration within an ameloblast could rise to many times that present in the extracellular matrix, leading to ameloblast cell stress.

Exposure to excess F^−^ can trigger endoplasmic reticulum (ER) stress within ameloblasts and compromise protein secretion [13], [14]. Secreted proteins pass through the ER. The ER functions as a quality control organelle and prevents misfolded proteins from traversing the secretory pathway [15]. Factors that adversely affect ER homeostasis cause ER stress and initiate an ER-to-nucleus signaling pathway, termed the unfolded protein response (UPR). Activation of the UPR results in transient attenuation of protein translation, enabling cells to cope with the existing protein load. The UPR also upregulates chaperones, augmenting the folding capacity of the ER. Accumulated proteins may also be removed via the ER-associated degradative pathway. UPR-mediated alleviation of ER stress may allow the cell to survive; prolonged ER stress can result in apoptosis [16], [17].

Here, we ask if low pH reduces the threshold dose required to induce F^−^ -mediated stress and if this stress results in decreased protein secretion. We also ask if rat incisor maturation stage ameloblasts that are naturally exposed to a low pH are more sensitive to F^−^-induced stress than secretory stage ameloblasts.

## Results

### Low pH enhances F^−^-mediated stress

**Figure 1:**
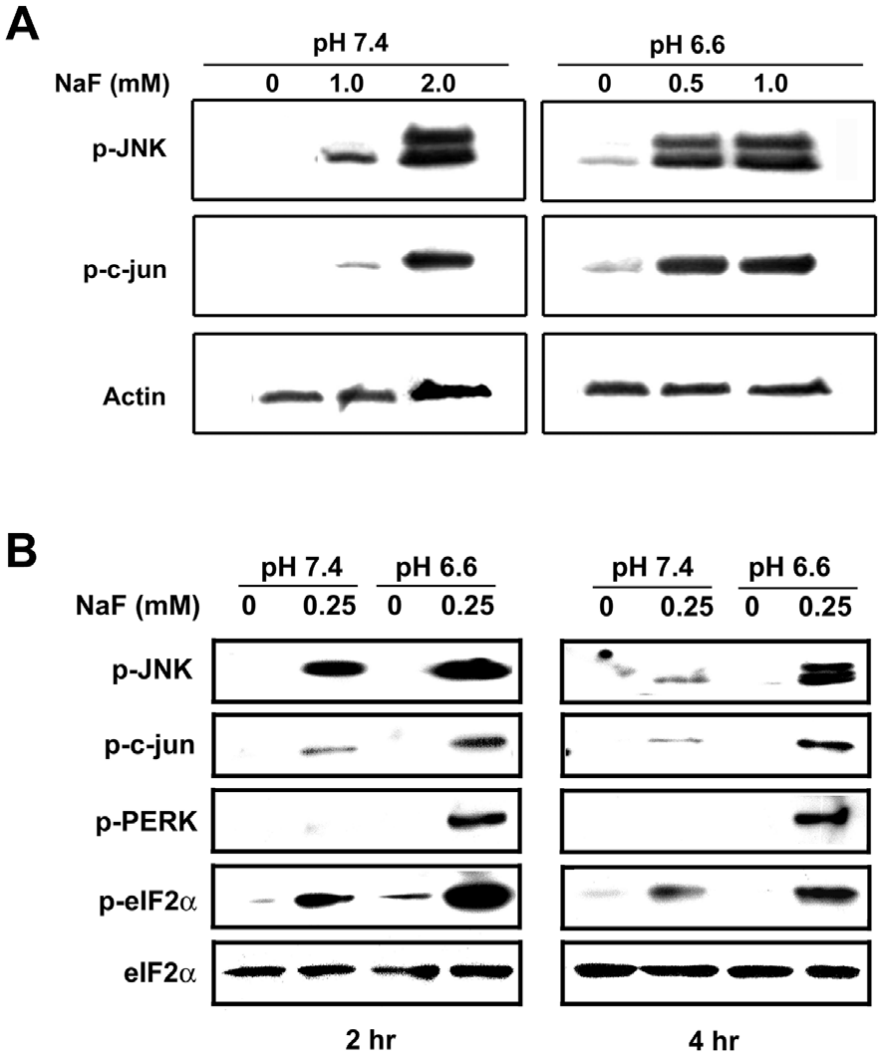
Low pH enhances F^−^-mediated stress. (A) Immunoblots of LS8 cells treated with indicated doses of NaF for 2 hr at pH 7.4 or pH 6.6 were probed for phosphorylated JNK and c-jun. Actin bands are controls for protein loading. (B) Immunoblots of LS8 cells treated with 0.25 mM NaF for 2 hr or 4 hr were probed for phosphorylated forms of JNK, c-jun, PERK and eIF2α. Total eIF2α bands are controls for protein loading. In all cases, low pH enhanced stress protein activation.

F^−^ can induce ER stress and activate the UPR in ameloblasts *in vivo* as well as in ameloblast-like LS8 cells *in vitro* [13], [14]. Activation of the UPR can result in the phosphorylation of JNK and c-jun [18], [19], [20]. To determine if low pH enhances F^−^ -mediated stress, we treated LS8 cells with F^−^ at pH 6.6 or pH 7.4 and monitored phosphorylation of JNK and c-jun. Both proteins were phosphorylated at higher levels at low pH as compared to treatment at neutral pH. The phosphorylation observed at 2 hr with 2.0 mM F^−^ at pH 7.4 were similar to that observed with 0.5 mM F^−^ at pH 6.6 (Figure 1A). In addition, F^−^ treatment at low pH consistently resulted in more phosphorylation of these proteins at all doses assayed (Figure 1B).

The serine/threonine kinase, PERK, is a primary sensor of the UPR that is activated by phosphorylation. Activated PERK phosphorylates the translation initiation factor, eIF2α, resulting in a transient attenuation of protein translation. This allows cells to cope with existing accumulated proteins within the ER. As shown in Figure 1B, exposure to F^−^ for 2 hr or for 4 hr at pH 6.6, relative to pH 7.4, enhanced PERK and eIF2α phosphorylation. Total levels of eIF2α reflect protein loading. Taken together, these results indicate that at low pH, lower doses of F^−^ are required to activate stress-related proteins.

### Low pH further decreases the F^−^-mediated reduction in protein secretion

During the secretory stage, ameloblasts secrete large amounts of proteins such as amelogenin, enamelin and the enzyme, MMP-20, that help form the organic matrix. During the maturation stage, ameloblasts secrete KLK4, a proteinase that helps in the degradation and resorption of the organic matrix. Therefore, protein secretion is a key function of ameloblasts that is essential for enamel formation. We have previously shown that F^−^ decreases protein secretion in a dose-dependent manner at neutral pH [13].

**Figure 2:**
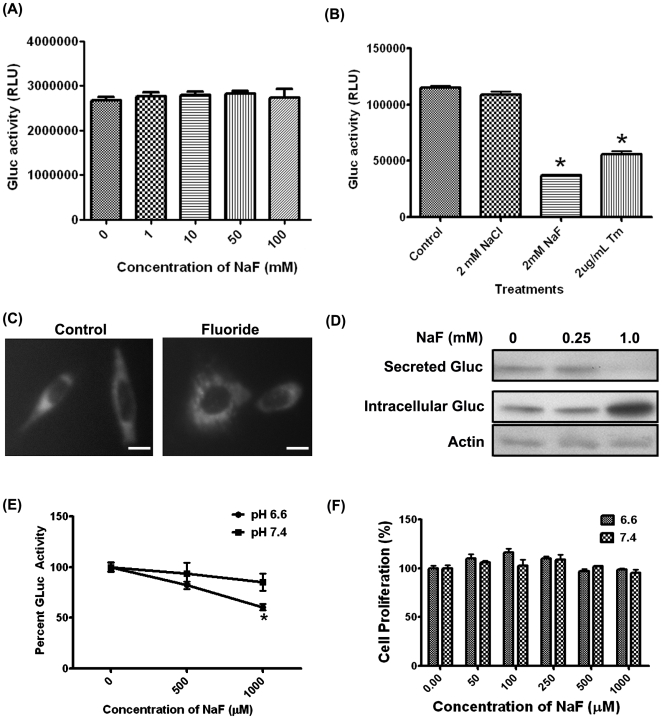
Low pH further decreases the F^−^-mediated reduction in protein secretion. (A) Recombinant Gluc was harvested from medium supernatant and directly treated with the indicated doses of NaF at 37°C for 6 hr. No significant decrease in Gluc activity was observed, demonstrating that F^−^,by itself does not inhibit Gluc activity (B) LS8-Gluc-CFP cells were treated with NaCl, NaF or the ER stress-inducing agent, tunicamycin for 6 hr; medium supernatant was then analyzed for Gluc activity (secretion). NaF and tunicamycin, but not NaCl, decreased Gluc secretion. (C) LS8-Gluc-YFP cells were treated with 0.25 mM NaF for 6 hr and imaged for YFP. NaF treatment localized the fusion protein within the peri-nuclear region. (D) LS8-Gluc-CFP cells were treated with the indicated doses of NaF for 24 hr and medium supernatants and cell lysates were immunoblotted and probed for Gluc. Actin served as the loading control. Note that F^−^ treatment resulted in intracellular accumulation of Gluc. (E) LS8-Gluc-CFP cells were treated with NaF at pH 6.6 or 7.4 for 2 hr. Gluc activity (secretion) in medium supernatant significantly decreased at pH 6.6 (p<0.05) (F) Cell proliferation, as measured by WST1 assay after 6 hr treatment, did not change significantly, indicating that the observed differences were not due to a proliferative advantage of one treatment group over another, in the short time period examined. All experiments were performed in triplicate and repeated three times. Scale bar for (C) represents 10 µm.

To determine if the F^−^-mediated decrease in protein secretion was further reduced by low pH, we stably transduced LS8 cells with either of two different secreted Gluc reporter constructs (LS8-Gluc-CFP or LS8-Gluc-YFP). Medium supernatant was assayed for Gluc activity. When recombinant Gluc was harvested and directly incubated with F^−^, no change in activity was observed (Figure 2A), demonstrating that F^−^ does not affect Gluc enzymatic activity. Exposure of Gluc-transduced LS8 cells to NaF decreased Gluc secretion, as assessed by Gluc activity in the culture medium. However, treatment with NaCl did not affect Gluc secretion, indicating that F^−^ but not Cl^−^ was toxic to the cells (Figure 2B). Tunicamycin, an agent that induces ER stress by inhibiting N-linked glycosylation, was used as a positive control.

In untreated controls, Gluc-YFP was present throughout the cell, presumably within the secretory pathway. In contrast, treatment with 0.25 mM NaF for 6 hr caused peri-nuclear accumulation of Gluc (Figure 2C). Immunoblots for Gluc showed that F^−^ caused a decrease in secretion and conversely, enhanced intracellular accumulation (Figure 2D). Together, these data indicate that F^−^ interfered with the secretion of Gluc and presumably, other endogenous secreted proteins, resulting in their intracellular accumulation.

To determine if low pH affected the F^−^-mediated decrease in protein secretion, we treated LS8-Gluc-CFP cells with F^−^ at pH 6.6 or 7.4 with 0.5 mM or 1 mM F^−^ (Figure 2E). A significant decrease in Gluc activity (p<0.05) at pH 6.6 was observed within 2 hr as compared to Gluc activity at pH 7.4. This decrease in Gluc activity could not be attributed to changes in cell proliferation (Figure 2F). Therefore, Gluc secretion in the presence of F^−^ was pH-dependent.

### Maturation stage ameloblasts experience higher levels of F^−^-induced stress

To confirm our cell culture results *in vivo*, we compared stress-induced phosphorylation levels of eIF2α in ameloblasts of rats drinking 0 or 100 ppm F^−^-treated water for 6 weeks. Rodent incisors grow continuously and therefore, are good models for studying fluorosis. However, a 10-fold higher F^−^ dose is required in rodents to induce plasma F^−^ levels equivalent to those found in humans [21]. This may be due to more efficient renal F^−^ clearance [22] and a shorter time-period of exposure to F^−^ during enamel formation. Rodent ameloblasts progress from the secretory stage to the final maturation stage in a matter of weeks whereas enamel development in humans may take years. F^−^ dose in rodents of 100 ppm is, therefore, representative of approximately 10 ppm in humans.

**Figure 3:**
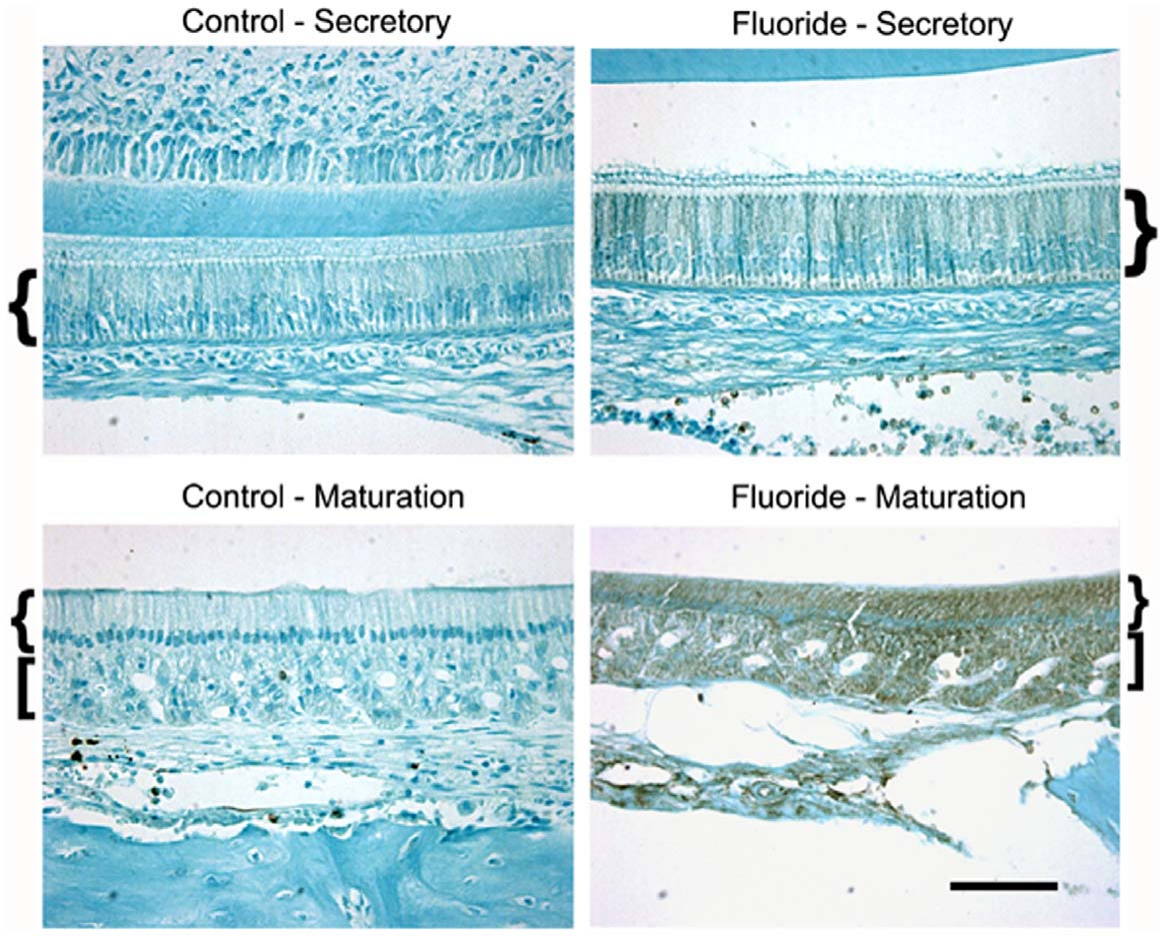
Maturation stage, but not secretory stage, ameloblasts from F^−^-treated rats exhibit stress. Rats were supplied *ad libitum* with 0 or 100 ppm F^−^ in their drinking water. Immunohistochemistry was performed on incisor sections with antiserum specific for phosphorylated eIF2α. Note significant staining in maturation stage ameloblasts and in the papillary layer but not in secretory stage ameloblasts of F^−^-treated rats. No staining was observed in the untreated rats. Curly brackets indicate ameloblasts and square brackets indicate papillary layer. Scale bar represents 50 µm.

Sagittal sections of the continuously growing rodent incisors reflect all ameloblast developmental stages. Therefore, effects of F^−^ on both secretory (pH∼7.2) and maturation stage ameloblasts (pH∼6.0) can be visually compared in the same rodent incisor. Staining for phosphorylated eIF2α was weak in secretory stage ameloblasts whereas staining was much more intense in maturation stage ameloblasts and in the surrounding papillary layer (Figure 3). No significant eIF2α phosphorylation was observed in ameloblasts from untreated control rats. These data suggest that the low pH environment of maturation stage ameloblasts sensitize them to the toxic effects of F^−^exposure.

### Maturation stage ameloblasts exhibit decreased gene expression

**Figure 4:**
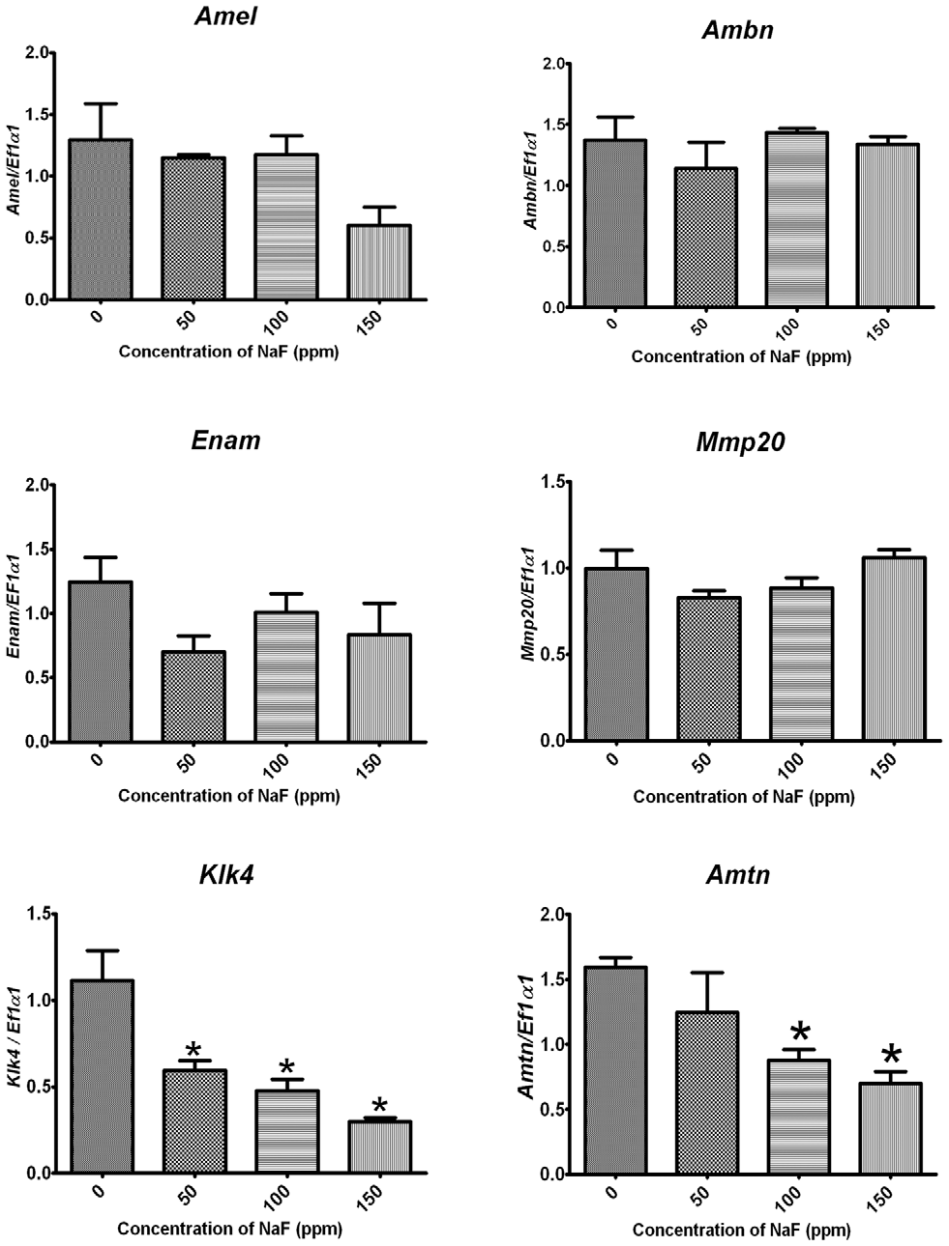
Decreased expression of maturation but not secretory stage-specific genes. Rats were treated with 0, 50, 100 or 150 ppm F^−^ in their drinking water for 6 weeks. qPCR was performed on secretory and maturation stage enamel organs. Data shown is an average of three separate experiments, performed in triplicate. Data was normalized to the *eEF1α1* expression control gene. Note the decreased expression of maturation stage genes, *Klk4* and *Amtn* (p<0.05). Secretory stage genes (*Amel*, *Ambn*, *Enam and Mmp20*) did not exhibit any changes in expression.

F^−^ toxicity can result in a decrease in mRNA expression *in vitro*. For example, a decrease in insulin mRNA was reported when beta-cells of the pancreas were exposed to 1.35 mM NaF [23]. Therefore, we asked if F^−^ decreased the expression of genes involved in enamel development and importantly, if the decrease occurred in a pH-dependent manner. Enamel matrix proteins, amelogenin (AMELX), ameloblastin (AMBN), enamelin (ENAM) and matrix metalloproteinase-20 (MMP20), are pre-dominantly secreted during the secretory stage at neutral pH. Conversely, the cell-adhesion protein, amelotin (AMTN), and the matrix-degrading enzyme, kallikrein-4 (KLK4), are secreted during the acidic maturation stage. Gene expression was quantified by qPCR in secretory and maturation stage enamel organs of incisors from rats treated with 0, 50, 100 and 150 ppm F^−^ *ad libitum* for 6 weeks. Expression levels of the secretory stage genes (*Amelx*, *Ambn*, *Enam* and *Mmp20*) were not reduced by F^−^ treatment (Figure 4). However, F^−^ treatment significantly reduced the expression of both maturation stage genes. Expression of *Klk4* decreased significantly at the lowest dose tested (50 ppm, p<0.05) and the expression of *Amtn* decreased significantly at 100 ppm F^−^. These data are consistent with reports indicating UPR-mediated degradation of mRNAs encoding proteins destined for secretion or for proteins that localize to the plasma membrane [24], [25]. Therefore, these results demonstrate F^−^ decreases enamel matrix gene expression and that this decrease occurs in the maturation stage, when the pH is acidic.

## Discussion

Enamel formation begins with the secretion of enamel matrix proteins during the secretory stage of enamel development. Together, these proteins form a matrix that organizes the hydroxyapatite crystals of the enamel. Once the crystals reach their full length, ameloblasts secrete KLK4 to degrade the matrix proteins, allowing the crystals to grow in width and thickness. The degraded proteins are then resorbed by ameloblasts, leaving behind fully mature, hardened enamel that has a mineral content greater than 96%. Compared to normal enamel, fluorosed enamel has a lower mineral content and a higher protein content [26], [27], [28], [29], [30], [31], [32] and therefore, has reduced hardness. Retention of the matrix proteins is thought to be responsible for the higher protein content of fluorosed enamel [26], [33], [34], [35], [36]. It was previously suggested that F^−^ decreases KLK4 activity, resulting in increased protein retention [28]. However, mechanisms leading to reduced KLK4 activity are not known.

Ameloblasts are unique because, during the maturation stage of enamel formation, they are in direct contact with the acidic mineralizing enamel matrix (pH<6.0) [8]. They are not as well-protected as other cells exposed to low pH, such as the cells lining the stomach. The latter are sheltered by a bicarbonate-rich mucus barrier that neutralizes the acid produced during digestion [37], and are continually replaced every 3–5 days. Ameloblasts, on the other hand, do not have any protective barriers and are not regenerated. Therefore, maturation stage ameloblasts may be directly exposed to F^−^ under low pH conditions.

Several reports point toward a relation between F^−^ and pH. For example, a decrease in pH facilitated the entry of F^−^ into L929 fibroblasts [38]. In addition, F^−^-mediated cytotoxicity in osteosarcoma cells was enhanced by low pH [39]. F^−^ uptake in micro-organisms also occurs as a function of the culture medium pH gradient [40], [41]; F^−^-resistant mutants become more sensitive to effects of F^−^ at low pH [42]. *In vivo*, F^−^ absorption rate from the stomach increased as the gastric pH decreased [43]. Similarly, a decrease in serum pH increased F^−^ absorption in the hamster cheek pouch and in the renal tubules of rat [44], [45], rabbit [46], dog [47] and human [48], [49], [50]. Conversely, less fluoride was excreted as the urinary pH decreased [45], [51]. Significantly, rats rendered acidotic by treatment with NH_4_Cl retain increased quantities of F^−^ in their dental enamel [52]. Therefore, the more acidic the extracellular fluid, the greater the tissue fluoride concentration [53], [54].

**Figure 5:**
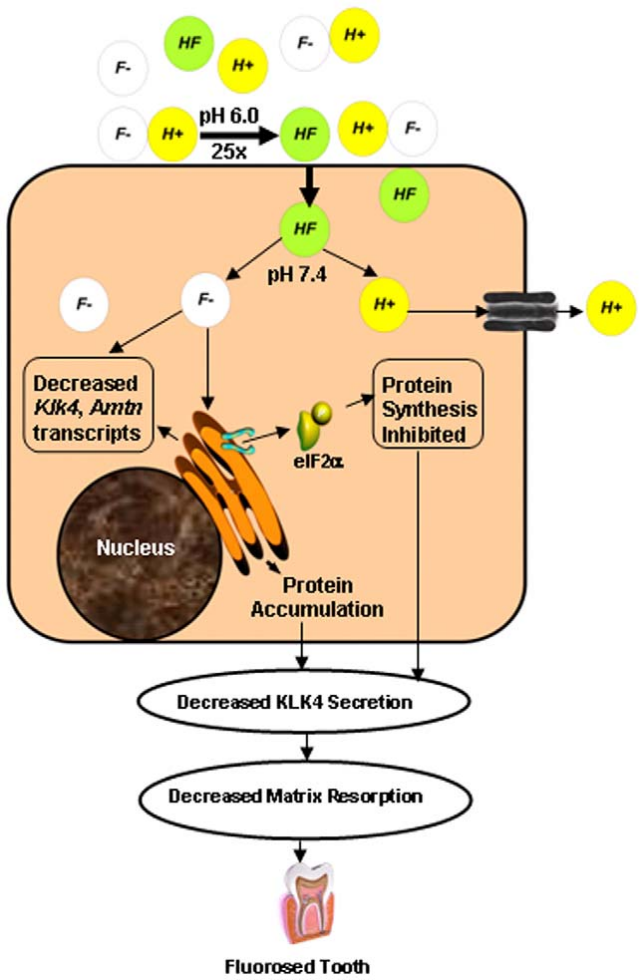
Schematic showing our postulated mechanism for maturation stage ameloblast sensitivity to fluoride. During the maturation stage, massive precipitation of hydroxyapatite occurs, releasing H^+^ ions. F^−^ can reversibly associate with H^+^ ions to form HF. Approximately 25-fold more HF is formed at pH 6.0 as compared to pH 7.4. HF diffuses into the cell more easily than F^−^ and flows down a steep concentration gradient from the acidic maturation stage enamel matrix into the neutral cytosol of the ameloblast. The neutral pH inside the cell causes reversion of HF to F^−^. Excess F^−^ within the cell interferes with ER homoestasis that may result in the dimerization and phosphorylation of PERK and its substrate, eIF2α. Consequently, protein synthesis is attenuated. ER stress can also lead to increased degradation of transcripts encoding secreted proteins such as *Klk4*. Collectively, decreased secretion of matrix-degrading enzymes such as KLK4 can lead to delayed resorption of enamel matrix proteins, resulting in the higher protein content observed in fluorosed enamel. ER, endoplasmic reticulum.

Here, we propose a novel, integrated mechanism based on pH and cell stress to explain the development of dental fluorosis. We hypothesize that F^−^ is converted to HF during the acidic maturation stage of enamel development and that HF flows down a steep pH concentration gradient from the enamel matrix into the ameloblast cytosol. The neutral pH inside the cell reverts HF to F^−^. Excess F^−^ within the cell interferes with ER homoestasis, inducing ER stress and activation of the UPR (Figure 5), resulting in compromised ameloblast function.

We validate our hypothesis by demonstrating that low pH enhanced F^−^-mediated stress *in vitro* and *in vivo*. Phosphorylation of eIF2α was observed in the papillary layer as well as in the maturation stage ameloblasts. The complete absence of staining in the control (untreated) maturation stage ameloblasts as well as the papillary layer suggests that the staining is specific. However, the results are not surprising. Maturation stage ameloblasts are in contact with the papillary layer near the basal terminal bars [55]. Ameloblasts and papillary layer cells are extensively interconnected by several large gap junctions [56]. The presence of numerous coated vesicles and also microvilli in the papillary cells suggest that they function similar to ameloblasts in the transport of ions, water and small nutrients during maturation [57]. Therefore, it is possible that fluoride ions within the ameloblast could reach the papillary cells through the gap junctions. This would result in papillary cell stress and consequently, lead to the phosphorylation of eIF2α. Moreover, carbon-dioxide produced within the ameloblasts during metabolism can lead to the formation of bicarbonate ions and hydrogen ions, catalyzed by carbonic anhydrases (as shown below):
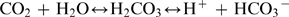
Ameloblasts contain at least 2 different carbonic anhydrases, CA2 and CA6 [58], [59]. Because the blood capillary-rich papillary layer is in close proximity with the ameloblasts, it is likely that the H^+^ ions are pumped to the capillaries and that this will cause a local decrease in the extracellular pH of the papillary layer as well.

We also showed that F^−^ inhibited cell function (Gluc secretion) in a pH-dependent manner. Indeed, F^−^-mediated decrease in protein synthesis and/or secretion has been well-documented [27], [28], [32], [60], [61], [62], [63], [64], [65], [66], [67], [68], [69], [70], [71], [72], [73]. Importantly, we demonstrated a decrease in enamel matrix transcripts during the maturation stage.

Taken together, our data show that F^−^ can regulate KLK4 activity by at least 3 different mechanisms. First, F^−^ can decrease KLK4 synthesis through stress-mediated phosphorylation of the translation initiation factor, eIF2α. This results in transient attenuation of global translation. Second, F^−^ can also decrease KLK4 secretion from ameloblasts. Third, F^−^ can decrease the steady state levels of mRNAs expressed during the maturation stage. While this can occur for all proteins that pass through the secretory pathway, it is especially important for *Klk4*. Reduced *Klk4* expression may hinder enamel matrix protein degradation and their removal. These mechanisms of F^−^ action provide an explanation for the higher protein content in fluorosed enamel as compared to normal enamel.

In conclusion, our research points toward a novel mechanism to explain fluorosis – namely, that the low pH environment of the maturation stage ameloblasts renders them more susceptible to F^−^ toxicity and that pH could be a defining factor in determining sensitivity of tissues to fluoride.

## Materials and Methods

*A complete methodology of experiments performed are listed in Supplementary Figure S1.*

### Ethics statement

All animals were treated humanely, based on a protocol approved by the Institutional Animal Care and Use Committee (IACUC) at The Forsyth Institute. The Forsyth Institute is accredited by the Association for Assessment and Accreditation of Laboratory Animal Care International (AAALAC) that follows the *Guide for the Care and Use of Laboratory Animals* (NRC1996).

### pH adjustment

Cell culture media containing 10% Fetal Bovine Serum (Invitrogen, Carlsbad, CA), were prepared using DMEM base lacking pH buffer (Sigma, St. Louis, MO), as described previously [74], [75]. NaHCO_3_ at 3 mM or 21 mM was added to the base to generate media with a pH of 6.6 or 7.4 respectively, in a 5% CO_2_ atmosphere. Medium osmolarity was adjusted by adding NaCl.

### Protein secretion assay

LS8 cells were transduced with lentiviral vectors expressing Gaussia luciferase (Gluc) under the control of a CMV promoter. Gluc was either indirectly tagged to Cerulean Fluorescent Protein (CFP) through an IRES element or directly fused to Yellow Fluorescent Protein (YFP) as described previously [76], [77]. Cells transduced with either construct demonstrated a decrease in protein secretion on exposure to fluoride. LS8-Gluc-CFP was used for protein secretion assays because the Gluc and CFP are translated as separate proteins, thereby avoiding any conflicts in post-translational modifications. LS8-Gluc-YFP was used to monitor the intracellular location of Gluc at a given timepoint by immunocytochemistry. LS8-Gluc-CFP and LS8-Gluc-YFP clones were isolated by flow cytometry. Protein secretion was determined as a function of Gluc activity. LS8-Gluc cells were seeded at a density of 25,000 cells / well in 6-well plates and treated with NaF at pH 6.6 or 7.4. Aliquots of 10 µL medium supernatant were mixed with 20 µM coelenterazine (Prolume Ltd./Nanolight, Pinetop, AZ) and the resulting bioluminescence measured for a 10 sec interval in a luminometer (Dynex, Richfield, MN). All experiments were performed in triplicate and repeated three times. Treated cell results were normalized to their untreated controls at their respective pH.

### Cell proliferation assay

LS8-Gluc-CFP cells were plated at a density of 2500 cells/well in 96-well plates. NaF-containing medium at pH 6.6 or 7.4 was added. Cell proliferation was determined after 6 hr by adding WST-1 (Roche Diagnostics, Mannheim, Germany) and measuring the resulting absorbance at 440 nm. All experiments were performed in triplicate and repeated three times. Treated sample values were normalized to control values at their respective pH and calculated as percent proliferation.

### Immunoblotting

To detect stress-related proteins, LS8 cells were treated with NaF at pH 6.6 or pH 7.4 for 2 hr or 4 hr. To determine the effect of F^−^ on secretion, LS8-Gluc-CFP cells were treated with NaF at pH 6.6 or pH 7.4 for 24 hr. Medium supernatant was assessed for secreted Gluc and lysates were assessed for intracellular Gluc. Cell lysates were prepared using Complete Lysis-M reagent containing protease and phosphatase inhibitors (Roche Diagnostics). Protein concentration was determined using the BCA assay kit (Pierce, Rockford, IL). Proteins (10–30 µg) were loaded onto 4–20% polyacrylamide gels (Biorad, Hercules, CA), transferred to nitrocellulose membranes (Schleicher and Schuell, Whatman, Germany) and probed with primary antibodies, as described previously [13]. Primary antibodies included: mouse anti-Gluc (Prolume Ltd./Nanolight); rabbit anti-eIF2α[pS^52^] and mouse anti-eIF2α (BioSource, Camarillo, CA); mouse anti-actin (Sigma); rabbit anti-phospho c-jun, rabbit anti-phospho PERK and rabbit anti-phospho-JNK (Cell Signaling, Danvers, MA).

### Real-time quantitative PCR (qPCR)

Six-week old rats were divided into 4 groups of three rats each and fed water containing 0, 50, 100 or 150 ppm F^−^, *ad libitum*. F^−^ concentration in water was confirmed using an F^−^ ion-selective electrode. All animals were treated humanely and with regard for alleviation of suffering. After 6 weeks, rats were sacrificed and secretory and maturation stage enamel organs were dissected from maxillary and mandibular incisors. RNA was extracted using Trizol™ (Invitrogen) and converted to cDNA (SuperScript III first-strand synthesis system, Invitrogen). All qPCR amplifications were performed as described previously [78]. Relative expression levels were calculated as a function of the internal reference control gene, e*EF1*α*1*. Primers used were: *AmelX*, ( *5′* TCATCCTGGGAGCC CTGGTTAT *3′* and *5′* GGCTGCCTTATCATGCTCTGGTA *3′* ); *Ambn* ( *5′* GGCCTGCTC CTGTTCCTGTCC *3′* and *5′* CTGCAAGCTTCCCAACTGTCTCATT *3′* ); *Enam* ( *5′* GGCT TTACCCCTATCAACAAC *3′* and *5′* TTCATAATCTTCAAACATCTCTTCTG *3′* ); *Mmp20* ( *5′* CACAGCTTTAAAGTTTGCCACTGC *3′* and *5′* GGGGGCCTCCTTTCTTTGTAT *3′* ); *Klk4* ( *5′* AGCCTGGCAGTCGGATGTTAGAG *3′* and *5′* GGAATGCGCCTGATGGTGTT AG *3′* ); *Amtn* ( *5′* CCTCCTTATCCACCCCTTGTTCC *3′* and *5′* GGGGTGCTCATTTCGT AGTCATCA *3′* ); and e*Ef1α1* ( *5′* TGATGCCCCAGGACACAGAGACT *3′* and *5′* GATAC CAGCTTCAAATTCCCCAACAC *3′* ).

### Immunocytochemistry and immunohistochemistry

To visualize the subcellular location of Gluc, LS8-Gluc-YFP cells were grown on 4-chamber tissue culture-treated glass slides (BD Biosciences, Bedford, MA) and treated with 0.25 mM NaF for 6 hr. Cells were fixed with 3% paraformaldehyde and imaged.

For immunohistochemistry, adult rats were treated with 0 or 100 ppm F^−^-containing water *ad libitum*. After 6 weeks, control and F^−^-treated rat incisors were extracted, fixed and embedded in paraffin. Sections were incubated with rabbit anti-phospho-eIF2α (BioSource), followed by incubation in peroxidase-conjugated antibody (Vectastain Elite Reagent, Vector Labs, Burlingame, CA) and in Sigma Fast 3,3′-diaminobenzidine substrate (Sigma). Sections were counterstained with 0.1% Fast Green in PBS and examined by light microscopy.

### Statistics

One-way ANOVA with Bonferroni post test was performed using GraphPad Prism version 5.00 for Windows (GraphPad Software, San Diego, CA). For analyzing significance of real-time PCR results, student's t-test was used. A *p*-value <0.05 was considered significant.

## Supporting Information

Figure S1 An outline of experiments performed.
(0.20 MB TIF)
