# Supplementary figures and images for: The Acid Test of Fluoride: How pH Modulates Toxicity
Source: PLoS One. 2010 May 28;5(5):e10895. doi: 10.1371/journal.pone.0010895 (PMC2878349; doi:10.1371/journal.pone.0010895)

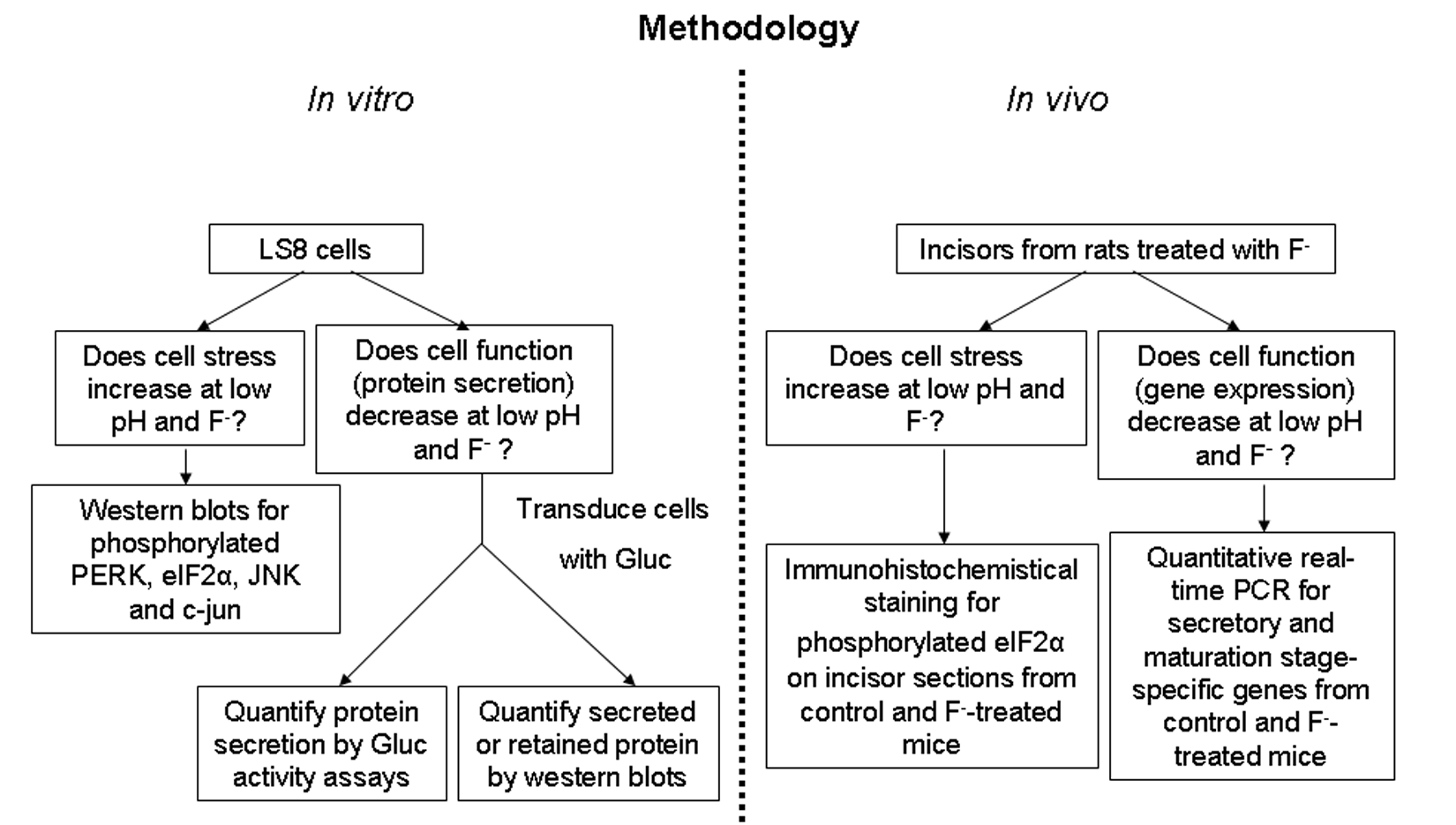

Supplement: Figure S1 — An outline of experiments performed. (0.20 MB TIF) [file pone.0010895.s001.tif]
